# Nursing Students’ Perceptions of a Novel Education Approach to Prevention and Control of Healthcare-Associated Infections: Insights from PrevInf Pilot Study

**DOI:** 10.3390/nursrep14020112

**Published:** 2024-06-14

**Authors:** Paulo Santos-Costa, Filipe Paiva-Santos, João Graveto, PrevInf Group

**Affiliations:** 1 Health Sciences Research Unit: Nursing (UICISA: E), Nursing School of Coimbra (ESEnfC), 3000-232 Coimbra, Portugal; paulocosta@esenfc.pt (P.S.-C.); jgraveto@esenfc.pt (J.G.); 2 Nursing Research, Innovation and Development Centre of Lisbon (CIDNUR), Nursing School of Lisbon, 1600-096 Lisboa, Portugal

**Keywords:** infection control, education, nursing, diploma programs, pilot projects

## Abstract

Background: Healthcare-associated infections (HAIs) pose a significant global threat, particularly in developing regions such as Southeast Asia. International bodies emphasize the role of formal undergraduate training in the prevention and control of HAIs. To address this, we aimed to explore the perceptions of Southeast Asian nursing students regarding a novel educational approach developed by a European–Southeast Asian project consortium. Methods: A pilot study was conducted in four nursing higher education institutions from Cambodia and Vietnam. First, local nursing educators conducted a 2 h classroom-based training session. Then, students were invited to participate for the first time in one of twelve evidence-based simulation scenarios developed by the research team, covering a range of nursing care situations related to the prevention and control of HAIs. After attending both components, students were asked to complete a paper-based questionnaire and rate their agreement with a set of statements on the appropriateness and meaningfulness of both components. Results: A total of 430 nursing students enrolled in the pilot study; 77.4% were female, with an average age of 19.8 years. The PrevInf educational intervention received positive feedback from participating students across settings, with strong agreement on the importance of proactiveness in competency development (*M* = 5.9, *SD* = 1.4). Notable differences between Cambodian and Vietnamese students were observed in terms of their receptiveness to the pre-selected teaching materials (*p* = 0.001) and strategies (*p* = 0.01) used by the nursing educators during their experience with the simulation scenarios. Conclusions: The PrevInf educational intervention shows promise in engaging Southeast Asian nursing students and fostering a deeper understanding of the prevention and control of HAIs. Further studies are warranted to refine the learning content and standardize the pedagogical strategies used by nursing educators across settings. This study was not registered.

## 1. Introduction

Healthcare-associated infections (HAIs) pose a persistent threat to global patient safety [1], particularly impacting low- and middle-income countries (LMICs) [2]. Research shows that among every 100 patients in healthcare settings, 7 from high-income countries and 10 from LMICs contract at least one HAI [3]. Healthcare-associated infections have consequences, such as: (i) increased morbidity and mortality; (ii) increased length of hospital stay; (iii) increased costs for health systems; (iv) reduced patient quality of life; (v) increased resistance to antimicrobials; and (vi) increased hospital readmissions [4,5,6,7]. The heightened vulnerability of the LMIC stems from a complex interplay of factors, including resource constraints, diverse healthcare settings, and varied educational landscapes.

International organizations and healthcare associations consistently emphasize the pivotal role of formal training and education in the prevention and control of HAIs as indispensable elements in developing a resilient healthcare workforce. In fact, recognizing the critical importance of preventing and controlling HAIs, the World Health Organization advocates for the integration of modules addressing these issues into undergraduate healthcare courses [8]. This integration aims to provide students with the necessary skills to actively contribute to enhancing clinical practice. Nevertheless, evidence of its effectiveness is still scarce [8], especially in LMICs.

The education and training of nursing students in infection prevention and control has several weaknesses and needs to be rethought and restructured in order to develop the skills necessary for the challenges posed by the prevention and control of HAIs [9]. The education and training of nursing students in this field are essential to ensuring the delivery of high-quality and safe care, as they play a crucial role in both avoiding pathogen transmission and preventing infectious diseases across various patient settings during clinical placements [10,11]. Despite the longstanding nature of this challenge, there remains an ongoing need for innovative approaches to enhance nursing students’ education and training in the prevention and control of HAIs [11,12]. It is essential to systematically integrate IPC (infection prevention and control) principles, along with modern education and training, into the curriculum in order to equip future nurses with the knowledge and skills necessary for adopting best-practice attitudes [13]. Education and training in the prevention and control of HAI, especially simulation-based training, can be an important complement to traditional educational strategies, improving students’ confidence in this topic, which is crucial for patient safety and developing resilience in healthcare professionals [14].

Tsioutis and colleagues have reported significant variability in the content, duration, recognition, and assessment of HAI prevention and control education across European countries [15]. Similar findings have been found in other across high-income countries such as Australia and Canada, as well as in LMICs such as Indonesia and Pakistan [16]. Similarly, Moghnieh and colleagues discovered that the majority of countries in the Eastern Mediterranean Region have fragmented and non-uniform HAIs education programs within human health undergraduate majors, and these programs lack recognition as standalone modules [17]. 

As far as we know, the curricula of nursing courses in Southeast Asia are not uniform in content, particularly in the way in which issues related to the prevention and control of HAIs are addressed, despite the region being recognized by the International Council of Nurses (ICN) as one of the primary sources for recruiting nursing professionals globally [18]. Despite the cultural, ethnic, and economic diversity among Southeast Asian nations, the prevention and control of HAIs pose a significant challenge throughout the region [19]. This emphasizes the critical need for standardized curricula and pedagogical approaches to guarantee uniform and comprehensive education in the prevention and control of HAIs for local nursing students.

Given the challenge mentioned, an international consortium of European and Southeast Asian higher education institutions (HEIs) was funded to enhance nursing curricula in the region, focusing on prevention and control of HAIs by assessing and updating the learning objectives, curriculum content, and educational methods and tools. This article aims to explore the perceptions of Southeast Asian nursing students regarding a novel educational approach developed by the consortium that includes a model-based training session and first-time participation in simulation scenarios.

## 2. Materials and Methods

An exploratory piloted study was simultaneously conducted in HEIs from Cambodia and Vietnam. In Cambodia, data collection took place at the International University (IU, located in Phnom Phenh) and Bolyno International Group (BNI, located in Phum Ou Rung—Kampong Chhang). In Vietnam, the educational intervention was implemented at Nam Dinh University of Nursing (NDUN, located in Nam Dinh) and Hai Duong Medical Technical University (HMTU, located in Hai Duong). The educational intervention was developed by the PrevInf Project consortium (as part of its activities funded by the Erasmus+ Agency through its Strategic Partnerships for Higher Education Programme (grant number: 618396-EPP-1-2020-1-PT-EPPKA2-CBHE-JP). The PrevInf consortium comprises the following universities: Nursing School of Coimbra (ESEnfC; project coordinating institution), Savonia University of Applied Sciences (European partner), and the HEIs previously identified (where the PrevInf model was implemented).

### 2.1. Sample and Recruitment

Recruitment occurred concurrently at all sites from January to February 2023. The study’s target population comprised undergraduate nursing students from IU, BNI, NDUM, and HMTU who voluntarily expressed interest in participating in the ongoing research. Inclusion criteria encompassed proficiency in either Vietnamese or Khmer, an age exceeding 18 years, and the provision of signed informed consent. Undergraduate students who engaged in short-term mobility actions affiliated with the participating universities were not included in this study.

### 2.2. Intervention

**Figure 1:**
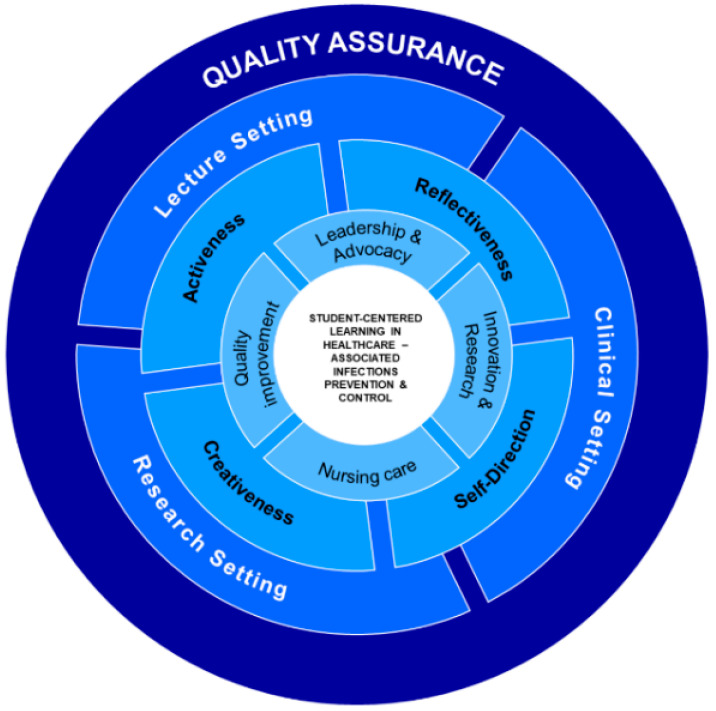
The PrevInf theoretical model.

The PrevInf educational intervention comprised two primary components: (i) the PrevInf training session (ii) and the PrevInf simulation scenarios. Regarding the first component, students were invited to participate in a 2 h theoretical lecture on the topic of prevention and control of HAIs. This lecture was conducted by local members of the PrevInf project. Students were initially presented with current epidemiological data and information on the challenges posed by this reality to patients, healthcare professionals, and healthcare organizations and systems. Then, the lecturer introduced the main objectives of the PrevInf project and emphasized the necessity for nursing students to develop their competencies in this field. The lecture followed the key elements of the PrevInf theoretical model (Figure 1), that is, the essential skills for the student to develop skills in preventing and controlling HAIs (leadership and advocacy, innovation and research, quality improvisation, and nursing care), the individual characteristics of students that influence the development of these skills (reflectiveness, self -direction, creativeness, and activeness), the importance of the contexts in which students develop their skills (lecture settings, clinical settings, and research settings), and the relevance of all these aspects to quality assurance in the prevention and control of HAIs.

Concerning the second component, the simulation scenarios developed by the PrevInf project consortium covered a range of nursing care situations related to the prevention and control of HAIs, based on evidence-informed recommendations from recent care standards and international guidelines. 

Nursing students participating in the educational intervention had the opportunity to engage with one of twelve distinct scenarios, each focusing on different care realities. Examples include the proper use of personal protective equipment (PPE) during the management of COVID-19 patients, prevention of intravenous line infections, assessment of infection risk during patient admission, and measures to prevent healthcare-associated pneumonia after an ischemic stroke. Other scenarios delved into topics such as hand hygiene, medication practices, aseptic procedures in the operating room, clinical waste management, needlestick injury prevention, environmental infection control, and respiratory tract infection prevention in tracheostomy patients. These scenarios collectively provide a comprehensive and hands-on approach to enhancing nursing students’ knowledge and skills in the prevention and control of HAIs.

Simulation scenarios took place in each university’s dedicated practicum laboratories. Following the methodology outlined by the International Nursing Association of Clinical Simulation and Learning [20], a conventional structure comprising a briefing (10 min), scenario (20 min), and debriefing (30–60 min) was implemented. Experienced nursing educators and researchers from the PrevInf Consortium, well-versed in this pedagogical method, assumed the roles of scenario instructors and simulated patients. Students were briefed on the scenarios beforehand and provided with carefully curated preparatory materials [21] encompassing both audio-visual and written resources, which were available in multiple languages and sourced from open-access platforms.

### 2.3. Study Variables and Instruments

Data were collected after students completed the educational intervention, which included both the theoretical aspect of the PrevInf training session and participation in a simulation scenario. Subsequently, students were instructed to complete a paper-based questionnaire and place it in a sealed box upon completion.

The questionnaire Nursing Students’ Perceptions of a Novel Education Approach to Prevention and Control of Healthcare-Associated Infections: Insights from PrevInf Pilot Study comprised three main sections. The first section consisted of 12 statements designed to explore how nursing students assessed their ongoing learning experiences in the field of HAI prevention and control, contrasting them with what they learned from the PrevInf training session. Students could rate their agreement with each statement on a 7-point Likert scale ranging from 1 (completely disagree) to 7 (completely agree), with a score of 4 considered neutral. The second section aimed to explore students’ attitudes or beliefs regarding the PrevInf simulation scenarios they participated in. This section comprised 11 statements that could be answered on a 5-point Likert scale ranging from 1 (completely disagree) to 5 (completely agree), with a score of 3 considered neutral. Finally, students were asked to complete a basic demographic questionnaire (e.g., age, sex, country, university, and course year).

### 2.4. Ethics

Approval for the research proposal under identification code 761-2021 (dated 5 April 2021) was granted by the Ethics Committee of the Health Sciences Research Unit: Nursing (UICISA: E) at the Nursing School of Coimbra (lead project partner). Before participating, all students provided informed consent, ensuring their voluntary participation in the study. Students received detailed information about the study’s objectives, educational methods, and their participant rights, including the option to withdraw without academic consequences. To maintain confidentiality, individual students were anonymized through coding, and data analysis was conducted collectively.

### 2.5. Statistical Analysis

Statistical analyses were performed using SPSS 26.0 for Windows (SPSS Inc., Chicago, IL, USA). Descriptive statistics, including mean, percentage, and standard deviation, were employed to characterize the study variables. The Kolmogorov–Smirnov test indicated that the data followed a normal distribution. Differences in questionnaire scores between countries were assessed using Student’s *t*-test for independent samples [22]. The significance level was set at ≤0.05. Questionnaires with missing data equal to or exceeding 20% were excluded from the analysis.

## 3. Results

A total of 430 nursing students enrolled in the pilot study from BNI (*n* = 143), HMTU (*n* = 111), IU (*n* = 110), and NDUN (*n* = 66). Most students were female (77.4%, *n* = 333), averaging 19.8 ± 1.7 (18–34) years of age. The most represented course year was the second (54%, *n* = 232), followed by the third (23.3%, *n* = 100) and first (22.3%, *n* = 96) course years. Only two fourth-year students participated in this study (0.5%). 

After the PrevInf training session, students were guided to assess their roles and ongoing learning experiences in the field of prevention and control of HAIs. Overall, students’ average scores indicate a medium to high level of agreement with the provided statements, despite significant score differences being found between Cambodian and Vietnamese students (Table 1).

Following their participation in one of the PrevInf simulation scenarios, students were invited to express their views on how it contributed to the enhancement of their knowledge and skills in the field. Students across the four partner HEIs provided favorable ratings for their recent simulation experiences (Table 2).

## 4. Discussion

After attending the PrevInf training session, students showed strong agreement with statements reflecting the importance of overseeing their development in HAI prevention and control by adopting a proactive attitude and exploring various opportunities to enhance competency in this field. Both Cambodian and Vietnamese nursing students acknowledged that their learning environment significantly influences the development of their competencies in this area, attesting to the importance of curricular improvement. 

Having infection-related content taught under multiple concepts with disparate exemplars can lead to potential gaps in the students’ competency related to the prevention and control of HAIs [23]. The PrevInf model-based training session introduced nursing students to key concepts for professional and academic development in this field, but also highlighted the need for transformation in their educational environment towards a more expansive, concept-based, and critically oriented training experience [24]. Addressing curricular fragmentation requires the creation of an educational environment that not only acknowledges, but actively promotes, a dialogical relationship among key stakeholders [17,25], including nursing educators, healthcare professionals, students, and health service users. This collaborative approach aims to facilitate not just a surface-level exchange of information, but a comprehensive and meaningful dialogue that addresses current teaching and learning challenges in this field faced by nursing HEIs in Southeast Asia.

Across the partner HEIs, nursing students emphasized the importance of peer learning and evidence-informed clinical practice in this field. Prior research has also emphasized the need for better alignment between theoretical education on prevention and control of HAIs in classrooms and practical experiences in clinical settings [26]. Education on the prevention and control of HAIs, along with peer role modeling, play crucial roles in shaping the behaviors of students and early nursing graduates. Previous authors have highlighted the significance of peer role modeling and its potential impact on repeated exposure to suboptimal practices in the prevention and control of HAIs by nursing tutors and other healthcare professionals [26]. Nursing students who witness such practices may become desensitized to proper prevention behaviors, potentially leading them to replicate observed substandard practices. Moreover, frequent exposure to these suboptimal practices during their clinical placements may lead to a perceived discrepancy between “what we are taught” and “how it is actually practiced”.

Remarkably, other notable differences in questionnaire scores were found, particularly regarding the perceived role of creativity in addressing challenges in this field. HAIs stand out as persistent safety challenges confronting healthcare professionals and organizations, a reality even more pronounced in times of heightened complexity in patient care and limitations in both human and clinical resources, such as the recent COVID-19 pandemic [27]. Within the healthcare workforce, nurses, being the most prominent professional group, are entrusted with the task of harnessing creativity alongside a methodological approach to address prevailing HAI issues [28]. This may involve introducing innovative medical devices, equipment, services, or strategic initiatives to effectively combat and manage these challenges. Despite the growing emphasis on innovation within nursing and healthcare in recent years, the integration of innovative concepts into nursing curricula is still in its early stages [28].

One of the most frequently referenced pedagogical strategies in international literature concerns simulation [10,29,30]. Simulation involves replicating authentic clinical settings to demonstrate procedures to students and enhance their competencies, such as decision making and critical thinking [10,31]. In our study, the nursing students’ first experiences with the simulation were overall positive, with moderate to high scores of agreement with the questionnaire statements. Overall, most nursing students identified simulation scenarios as critical learning opportunities that assist them in mastering specific HAI-related topics in a safe environment before having the opportunity to perform them in clinical placements.

Nevertheless, statistically significant differences were found between students from Cambodian and Vietnamese HEIs, with the former presenting lower scores of agreement with statements concerning the selected teaching materials, instructions, and strategies used by the nursing educators. These results may explain why Cambodian students felt less confident than Vietnamese students in mastering the specific topics targeted in the simulation scenarios (*t*_(428)_ = −2.81, *p* = 0.005). While these findings may result from cultural differences between the two countries, further iterations are warranted to ensure a standardized approach among nursing educators across Southeast Asian partner HEIs and similar levels of meaningfulness and appropriateness for nursing students.

### Limitations

Despite the positive outcomes observed in our study, it is important to acknowledge certain limitations. Firstly, a more comprehensive study design is necessary to investigate students’ perceptions of the educational intervention implemented in both Vietnam and Cambodia. Future research efforts should focus on the prospective implementation of educational interventions, employing repeated measures and conducting specific analyses of the effects across different course years (both at an undergraduate and postgraduate level). Thus, further research could be conducted to explore the effectiveness of different educational approaches in terms of improving students’ competence in the prevention and control of HAIs.

Moreover, despite receiving positive feedback from students in two different countries, it is essential to expand the involvement of partner HEIs from additional Southeast Asian countries such as the Philippines, Thailand, or Singapore. This expansion aims to iteratively refine the educational intervention, considering the diverse cultural, ethnic, and economic variations among countries. Lastly, we propose adopting a mixed-methods approach incorporating both quantitative and qualitative data. This approach will provide valuable insights to the project team, aiding in the further refinement and improvement of the intervention’s components.

## 5. Conclusions

The PrevInf educational intervention, encompassing the training session and simulation scenarios, garnered strong approval from the participating students. Following the presentation of the PrevInf model and the training session, nursing students displayed unanimous agreement with statements emphasizing the need for proactiveness in developing their competencies in the field. This extended to planning, delivering, and assessing interventions based on a creative, evidence-informed, critical, and reflexive practice. Students expressed high levels of satisfaction with the developed simulation scenarios, underscoring their pedagogical potential in enhancing knowledge and skills in the field of HAIs prevention and control. This positive response reflects the potential of the PrevInf educational intervention, not only in capturing the students’ engagement, but also in fostering a deeper understanding of the subject matter.

## Figures and Tables

**Table 1:** Students’ assessments of their ongoing learning experiences in the field of HAI prevention and control (Cambodia, *n* = 253; Vietnam, *n* = 177; Likert scale ranging from 1 (completely disagree) to 7 (completely agree)).

After Being Introduced to the PrevInf Model, How Do You Rate the Following Statements?	Cambodia *M* ± *SD (Min–Max)*	Vietnam *M* ± *SD (Min–Max)*	Student’s *t*-Test
I oversee my development in the field	5.5 ± 1.2 (1–7)	5.9 ± 1.8 (1–7)	*t*_(427)_ = −2.77, *p* = 0.006
My practice must be informed by the best available evidence in the field	5.5 ± 1.3 (1–7)	5.9 ± 1.7 (1–7)	*t*_(427)_ = −2.18, *p* = 0.030
Peer learning is important to develop my skills and knowledge in the field	5.6 ± 1.3 (1–7)	6.0 ± 1.8 (1–7)	*t*_(427)_ = −2.60, *p* = 0.010
Critical thinking is an essential ability for my development in the field	5.7 ± 1.1 (1–7)	5.9 ± 1.8 (1–7)	*t*_(427)_ = −1.02, *p* = 0.307
It is my responsibility to develop my competencies in this field	6.0 ± 1.0 (1–7)	6.0 ± 1.7 (1–7)	*t*_(426)_ = −0.21, *p* = 0.833
Creativity plays a fundamental role in tackling challenges in this field	5.5 ± 1.2 (1–7)	6.0 ± 1.8 (1–7)	*t*_(427)_ = −3.26, *p* = 0.001
I must explore different learning sources to develop my own competencies in this field	5.9 ± 1.1 (1–7)	6.0 ± 1.7 (1–7)	*t*_(426)_ = −0.57, *p* = 0.571
It is important to reflect on current practices to deliver quality care	5.9 ± 1.1 (1–7)	6.0 ± 1.7 (1–7)	*t*_(426)_ = −1.13, *p* = 0.261
I value the importance of evidence-informed practice in this field	5.8 ± 1.0 (2–7)	6.0 ± 1.7 (1–7)	*t*_(425)_ = −1.88, *p* = 0.061
I must adopt a proactive attitude and explore different opportunities to improve my competencies in this field	5.9 ± 1.1 (1–7)	6.0 ± 1.7 (1–7)	*t*_(427)_ = −0.62, *p* = 0.537
My ability to innovate can lead to better care outcomes for patients in this field	5.8 ± 1.1 (2–7)	6.0 ± 1.8 (1–7)	*t*_(427)_ = −1.07, *p* = 0.285
My learning environment plays a fundamental role in the development of my competencies in the field	5.8 ± 1.1 (1–7)	6.0 ± 1.7 (1–7)	*t*_(427)_ = −1.42, *p* = 0.157

**Table 2:** Students’ assessment of experience with the PrevInf (Cambodia, *n* = 253; Vietnam, *n* = 177; Likert scale ranging from 1 (completely disagree) to 5 (completely agree)).

Concerning Your Experience with the PrevInf Simulation Scenarios…	Cambodia *M* ± *SD (Min–Max)*	Vietnam *M* ± *SD (Min–Max)*	Student’s *t*-Test
The teaching methods used were helpful	4.17 ± 0.65 (1–5)	4.20 ± 1.28 (1–5)	*t*_(428)_ = −0.31, *p* = 0.761
It provided me with a variety of learning materials to promote my learning in the field of HAIs prevention and control	3.95 ± 0.74 (1–5)	4.22 ± 1.28 (1–5)	*t*_(428)_ = −2.55, *p* = 0.012
I enjoyed how my instructor taught the scenario	4.14 ± 0.63 (1–5)	4.23 ± 1.25 (1–5)	*t*_(428)_ = −0.88, *p* = 0.382
The teaching materials used were motivating	3.83 ± 0.77 (1–5)	4.17 ± 1.25 (1–5)	*t*_(428)_ = −3.22, *p* = 0.001
The way my instructor(s) taught the simulation was suitable to the way I learn	3.96 ± 0.72 (1–5)	4.23 ± 1.24 (1–5)	*t*_(428)_ = −2.60, *p* = 0.01
I am confident that I am developing my skills in the covered HAIs-related topic	4.17 ± 0.63 (1–5)	4.12 ± 1.20 (1–5)	*t*_(428)_ = 0.50, *p* = 0.616
I am confident that I am obtaining the required knowledge in the covered HAIs-related topic	4.05 ± 0.58 (1–5)	4.16 ± 1.21 (1–5)	*t*_(428)_ = −1.13, *p* = 0.26
I am confident that I am mastering the content of the simulation activity that my instructors presented to me	3.83 ± 0.74 (1–5)	4.12 ± 1.23 (1–5)	*t*_(428)_ = −2.81, *p* = 0.005
The simulation offered a variety of ways in which to learn the material	4.03 ± 0.68 (1–5)	4.21 ± 1.24 (1–5)	*t*_(428)_ = −1.73, *p* = 0.085
This simulation offered a variety of ways to assess my learning	4.07 ± 0.68 (1–5)	4.16 ± 1.24 (1–5)	*t*_(428)_ = −0.09, *p* = 0.367
This simulation scenario was critical for the mastery of the specific HAIs-related topic during my clinical practice	4.11 ± 0.72 (1–5)	4.23 ± 1.22 (1–5)	*t*_(428)_ = −1.18, *p* = 0.238

## Data Availability

The data presented in this study are available upon request from the corresponding author.

## Public Involvement Statement

No public involvement in any aspect of this research.

## Guidelines and Standards Statement

This manuscript was drafted against the Strengthening the Reporting of Observational Studies in Epidemiology (STROBE) for cross-sectional studies [32]. A complete list of reporting guidelines can be accessed via the equator network: https://www.equator-network.org/ (accessed on 3 January 2024).
